# Randomized, prospective, multicenter trial assessing the numen coil embolization system in the endovascular treatment of small intracranial aneurysms: outcomes from the CATCH Trial

**DOI:** 10.1186/s12893-023-02049-9

**Published:** 2023-06-16

**Authors:** Yazhou Jin, Xinbin Guo, Tao Quan, Rui Zhao, Tianxiao Li, Zhenwei Zhao, Hua Yang, Xingen Zhu, Guobiao Liang, Bing Leng, Xin Wu, Yang Wang, Sheng Guan

**Affiliations:** 1 Department of Neurointervention, Zhengzhou University First Affiliated Hospital, No.1, Jianshe East Road, Erqi District, 450052 Zhengzhou, Henan, China; 2 Department of Neurovascular Center, Changhai Hospital Affiliated to the Naval Medical University, Shanghai, China; 3 Department of Interventional Radiology, Henan Provincial People’s Hospital, Zhengzhou, Henan China; 4 Department of Neurosurgery, Tangdu Hospital Affiliated to Fourth Military Medical University, Baqiao, Xi’an, Shaanxi China; 5 Department of Neurosurgery, Affiliated Hospital of Guizhou Medical University, Yunyan, Guiyang City, Guizhou China; 6 Department of Neurosurgery, The Second Affiliated Hospital of Nanchang University, Nanchang City, Jiangxi Province China; 7 Department of Neurosurgery, The General Hospital of Shenyang Military, Shenhe, Shenyang, China; 8 Department of Neurosurgery, Huashan Hospital, Shanghai, China; 9 Department of Neurosurgery, Yantai Yuhuangding Hospital, Zhifu, Yantai, Shandong China; 10 Department of Neurosurgery, The First Affiliated Hospital of Nanchang University, Nanchang, Jiangxi China

**Keywords:** Numen Coil Embolization System, Endovascular treatment, Small intracranial aneurysms

## Abstract

**Background and purpose:**

The CATCH (Coil Application Trial in China) trial was designed to assess the safety and efficacy of the Numen Coil Embolization System in the treatment of intracranial aneurysms in comparison with the Axium coil (ev3/Medtronic). Although the endovascular treatment of small (< 5 mm) intracranial aneurysms has been reported with favorable long-term clinical and angiographic outcomes, randomized trials are still lacking. Data for aneurysms smaller than 5 mm were extracted from the CATCH trial.

**Materials and methods:**

A randomized, prospective, multicenter trial was conducted at ten centers throughout China. Enrolled subjects with small intracranial aneurysms were randomly assigned to receive treatment with the Numen Coil or the Axium coil. The primary outcome was successful aneurysm occlusion at the 6-month follow-up. In contrast, the secondary outcomes included complete aneurysm occlusion, recurrence rate, clinical deterioration, and safety data at the 6-month and 12-month follow-ups.

**Results:**

A total of 124 patients were enrolled in the study. Overall, 58 patients were assigned to the Numen group, and 66 were assigned to the Axium group. At the 6-month follow-up, the successful aneurysm occlusion rate was 93.1% (54/58) in the MicroPort NeuroTech group and 97.0% (64/66) in the Axium group, with a common odds ratio of 0.208 (95% confidence interval, 0.023–1.914; P = 0.184). Complications were comparable between the groups.

**Conclusions:**

Compared with the Aixum coil, the Numen coil is safe and effective in treating small intracranial aneurysms.

**Trial registration:**

(13/12/2016, NCT02990156)

## Introduction

Due to the unique structures of small aneurysms, technical difficulties exist in the treatment of small aneurysms, including ruptured and unruptured aneurysms. Compared with clipping, intravascular embolization has lower mortality and disability rates [1, 2]. Small ruptured aneurysms have been reported to account for approximately 50% of subarachnoid hemorrhage (SAH) cases [3, 4]. It has been reported that the intraprocedural rupture rates of small aneurysms are 2–5 times higher than those of more giant aneurysms [5, 6]. These difficulties, including the small size of the aneurysm sac, the stability of the embolization catheter, the reliability of coil detachment, and the conformability of coiling, increase the risk of rupture during the filling process [7, 8].

In recent years, with the development of manufacturing technology, the performance of coil delivery has been optimized. At the same time, to reduce the recanalization rate and enhance the durability of endovascular treatment, various materials have been added, including hydrogels, polyglycolic acid, and nylon.[9, 10]. Compared with bare platinum coils, these modified coils have exhibited a lower packing density and a similar safety profile in several studies [11–13]. However, these enhanced bioactive coil devices have not demonstrated sustained benefits. Multiple large prospective trials have revealed the safety and efficacy of bare platinum coils. The Axium coil (ev3/Medtronic) is a widely used bare platinum coil [14].

The Numen Coil Embolization System (MicroPort NeuroTech, Shanghai, China) is a new electric detachable bare-metal coil made of platinum-tungsten alloys. Preclinical studies have shown that it promotes thrombosis in the aneurysm sac and vascular endothelialization in the neck to treat intracranial aneurysms. Compared with other types of spring coils, there are various coils with small sizes and lengths, which ensure the safety of the endovascular treatment of small aneurysms.

In addition to the invention of appropriate spring coils, auxiliary techniques, such as stent-assisted and balloon-assisted coiling, provide patients with a minimally invasive and effective option. Compared with coiling-only methods, stent-assisted coiling techniques show similar immediate occlusion rates, lower recurrence rates, long-term angiographic occlusion rates, and higher ischemic stroke and mortality rates [15–17].

Many studies on the endovascular treatment of small aneurysms have been reported [3, 7, 15–17]. However, there are a series of shortcomings in these studies. First, most of them are retrospective studies with small sample sizes and fewer than 100 cases. Second, some studies were published five years ago or even earlier. Currently, intravascular treatment is relatively safe and feasible. Third, stent-assisted and balloon-assisted coiling techniques are commonly used in addition to coiling-only methods. A recent meta-analysis by Yamaki included 22 studies and 1105 small aneurysms but included only 86 cases of stent-assisted coiling [18]. Fourth, some studies have focused only on either ruptured or unruptured tiny aneurysms. In 2017, the lack of prospective data prompted us to lead a multicenter, randomized, controlled trial assessing the safety and efficacy of the endovascular treatment of small aneurysms. Compared with a well-established coil, i.e., the Axium coil, the CATCH (Coil Application Trial in China) trial was designed to assess the safety and efficacy of the Numen Coil Embolization System (MicroPort NeuroTech, Shanghai, China) in the treatment of all intracranial aneurysms. Data of aneurysms smaller than 5 mm were extracted from the CATCH trial.

## Materials and methods

### Study design

This study is a subgroup analysis of Catch data obtained from a prospective, multicenter, open-label, randomized controlled trial that includes patients treated with the Numen and Axium coil. The CATCH clinical trial includes patients treated with the Numen or Axium coil starting in August 2017 and concluding in December 2019, following the enrollment of 350 patients. The data of 140 patients whose aneurysms were smaller than 5 mm were extracted from the CATCH trial in this subgroup analysis. Details regarding the institutional review board and ethics committee approvals, patient population, and protocol requirements are described in the primary CATCH article.

This subgroupanalysis was performed to assess the safety and efficacy of the Numen coil Embolization System in treating of small intracranial aneurysms (< 5 mm) compared with the Axium coil. Data for analysis were basic demographic information, including patient age, sex, and history of aneurysm rupture; aneurysm characteristics, including aneurysm size and location; procedural data, including packing density, devices used; and follow-up data, including any complications. Before commencing recruitment, the study was registered with the China Clinical Trial Registry (NCT02990156).

### Participants

At each clinical center, eligible patients were screened if they met any of the following criteria: The patient was aged 18 to 80; ruptured (World Federation of Neurologic Societies grade < IV) or unruptured intracranial aneurysm, as demonstrated by computed tomography angiography (CTA), magnetic resonance angiography (MRA) or digital subtraction angiography (DSA); complete aneurysm cure with one treatment; the diameter was < 5 mm; and written formed consent before enrollment. Ineligible patients were excluded according to the following criteria: any contraindications for endovascular treatment, angiography, or anesthesia; severe stenosis or occlusion in the parent vessel; and the presence of a cerebral arteriovenous malformation or an intracranial lesion.

### Randomization and masking

All eligible patient allocations were randomized at a 1:1 ratio based on an interactive Web response system. Information technology specialists developed the system from an independent clinical research organization. The online central randomization method ensured that the allocation was concealed. In addition, minimization criteria were applied to balance possible factors, including neck width, diameter length, location, and rupture status. Masking the treatment allocation to the investigators was not possible. However, members who followed up with trial subjects were blinded to the assignment.

### Procedures

All recruited participants performed routine preoperative examinations to reduce surgical complications. All procedures were performed under general anesthesia and via a transfemoral approach. After sheath placement, systematic heparinization was individually administered to maintain an activated clotting time of 250–350 s. Then, appropriate coils were implanted into the aneurysm sac through a microcatheter. It was suggested that the test or control group coil length used in each aneurysm should be more than 50% of the total length. For wide-necked aneurysms, remodeling balloons or stents were recommended as assist devices, excluding flow diverters and covered stents. The antiplatelet and anticoagulation regimens were left to the individual operator, according to the standard of each clinical center. The drug usage of each participant was recorded in detail from preoperatively to 12 months postoperatively.

### Clinical and radiological assessments

All participants underwent preoperative preparation, including clinical examination and angiographic assessment of the target aneurysm. Before randomization, the parameters collected included age, sex, and rupture status. In addition, other baseline data were obtained on aneurysm size and location. After surgery, the following data were collected on the coils, assist devices, complications, and angiographic images. At 1-month post-implantation, investigators followed up with each participant by telephone. DSA was recommended at the 6-month follow-up, while MRA was suggested at the 12-month follow-up. In addition, complications and additional interventions for the target aneurysm at any time were documented.

Digital copies of imaging data before treatment, immediately after treatment, at the 6-month follow-up, and the clinical research organization collected during the 12-month follow-up. An independent core laboratory and Clinical Events Committee (CEC), comprised of three members qualified in neuroimaging or equivalent qualifications, located at the Sixth People’s Hospital, Shanghai, reviewed all the imaging and endpoint events. The degree of aneurysm occlusion was assessed according to the 3-class Raymond scale (complete occlusion, neck remnant, and residual aneurysm). The modified Rankin Scale (mRS) score was also obtained during the follow-up.

### Outcomes

The trial’s primary outcome was the proportion of participants with successful aneurysm occlusion at the 6-month follow-up. Members of the independent core laboratory reviewed the digital copies of the imaging data and assessed the degree of aneurysm occlusion. Successful aneurysm occlusion was defined as Raymond class I or II. The secondary outcomes included the following: immediately successful aneurysm occlusion rate, complete aneurysm occlusion at the 6-month and 12-month follow-ups (complete aneurysm occlusion was defined as no contrast filling in either the sac or the neck of the aneurysm); recurrence rate at the 6-month and 12-month follow-ups; clinical deterioration rate at 6-month and 12-month follow-ups (clinical deterioration was defined as an mRS score > 2 and an increase of at least 1 point); and safety data at the 1-month, 6-month and 12-month follow-ups (including adverse events, such as ischemic stroke, intraoperative or delayed aneurysm rupture, and neurological sequelae).In addition, the total coil length and coil packing density were also recorded. The coil packing density was defined by dividing the coil volume by the aneurysm volume [19]. Coil volume was determined using the formula V = π (d / 2)^2^ L, where d is coil diameter, and L is coil length. Aneurysm volume was calculated using the formula V = 4/3 π a b (a + b) /  2, where a and b are half of the long and short axes of the aneurysm.

### Statistical analysis

The baseline characteristics of patients between groups were compared to verify intergroup differences. Medians with interquartile ranges were used for continuous variables, and proportions were used for categorical variables. The t-test or Mann-Whitney U test was used for continuous variables, and the χ2 test or Fisher exact test was used for categorical variables. The primary and secondary outcomes were studied using odds ratios with a 95% confidence interval, and the χ2 test or Fisher exact test was used for comparisons between groups. The number of adverse events was compared by using Fisher exact test. All analyses were performed using SPSS version 23 (IBM, Armonk, New York). All tests were two-sided, and P < 0.05 was considered statistically significant.

## Results

### Baseline results

Three hundred and fifty patients were recruited into the CATCH trial between April 2017 and October 2017 under randomization. Recruitment was stopped after the target number of patients had been recruited. The data of 140 patients whose aneurysms were smaller than 5 mm were extracted from the CATCH trial. The 140 patients formed the full analysis set (FAS) and underwent the treatments of interest. Due to the small size of the aneurysm sac in one ruptured aneurysm case, investigators failed to deliver coils into the sac. Among the 140 treated patients, 14 withdrew from the study without reason, non-conformance of one subjects with inclusion and exclusion criteria, and non-usage of coils in one subjects according to their random allocation. The remaining 124 patients formed the per-protocol set (PPS). The baseline characteristics of the patients and the aneurysms are shown in Table 1. Fifty-eight patients (46.77%) in the analysis population were assigned to the Numen group, and 66 patients (53.23%) were assigned to the Axium group. The two groups were comparable. There were no significant differences in baseline characteristics between the two groups in the per-protocol set.

**Table 1:** Baseline characteristics of the per-protocol set

Characteristics	Numen N = 58	Axium N = 66
Sex ratio (male/female)	20:38	26:40
Mean age (year)	56 (9.89)	55 (9.57)
Aneurysm location		
Anterior circulation	57 (98.2%)	65 (98.4%)
Posterior circulation	1 (1.8%)	1 (1.6%)
Aneurysm size (mm)	3.85 (0.60)	3.83 (0.82)
Ruptured aneurysm	12 (20.7%)	19 (28.8%)
Unruptured aneurysm	46 (79.3%)	47 (71.2%)
Packing density (median) (%)	46.2 (35.6–54.3)	45.5 (33.3–60.5)
Total coil length (mm)	18.2 (10.79)	21.3 (9.75)
Assisted device used		
None	22 (47.4%)	29 (43.9%)
Balloon, no stent	4 (6.9%)	1 (1.5%)
Stent, no balloon	30 (51.7%)	36 (54.6%)

Data are No. (%), mean (SD), or median (interquartile range)

### Numen arm

Among patients allocated to the Numen group, the mean age was 56 ± 9.89. Twenty (34.48%) patients were male, while 38 (65.52%) were female. The mean aneurysm size was 3.85 ± 0.60 mm. Fifty-seven aneurysms (98.28%) were located in the anterior circulation, and 1 (1.72%) was found in the posterior circulation. Twelve patients (20.69%) were treated for ruptured aneurysms. Twenty-four patients (41.38%) were treated without using assistive devices. Stent-assisted coiling alone was used in 30 patients (51.72%), and balloon remodeling alone was used in 4 patients (6.90%). The mean packing density was 46.23 ± 23%, and the mean total coil length was 18.20 ± 10.97 mm.

### Axium arm

Among patients allocated to the Axium group, the mean age was 55 ± 9.57 years. Twenty-six (39.39%) patients were male, while 40 (60.61%) were female. The mean aneurysm size was 3.83 ± 0.82 mm. Sixty-five aneurysms (98.48%) were located in the anterior circulation, and 1 (1.52%) was in the posterior circulation. Nineteen patients (28.79%) were treated for ruptured aneurysms. Twenty-nine patients (43.94%) were treated without using assistive devices. Stent-assisted coiling alone was used in 36 patients (54.55%), and balloon remodeling alone was used in 1 patient (1.52%). The mean packing density was 45.50 ± 26%, and the mean total coil length was 21.29 ± 9.75 mm.

### Primary and secondary outcomes

Treatment and follow-up details are shown in Table 2. On the 6-month angiographic follow-up, the two groups showed no significant difference in the successful aneurysm occlusion rate. In the per-protocol analysis of 124 subjects for the primary outcome, the successful aneurysm occlusion rate was 93.1% (54/58) for the Numen group and 97.0% (64/66) for the Axium group, with a standard odds ratio of 0.208 (95% confidence interval, 0.023–1.914; P = 0.184). Similarly, the immediately successful aneurysm occlusion rate did not differ significantly between the two groups. The calculated technical success rate was 87.9% (51/58) and 93.9% (62/66) in the Numen group and the Axium group, respectively, with a standard odds ratio of 0.470 (95% CI, 0.130–1.696, P = 0.345). At the 6-month and 12-month follow-ups, there was no significant difference between the groups in the complete aneurysm occlusion rate or the recurrence rate. Patients with recurrence were not retreated and were told to check in regularly.

**Table 2:** Primary and secondary outcomes evaluation

Outcomes	Numen N = 58	Axium N = 66	Odds ratio (95% Cl)	P value
Success occlusion rate (6 M)	54 (93.1%)	64 (97.0%)	0.208 (0.023–1.914)	0.334
Immediate success occlusion rate	51 (87.9%)	62 (93.9%)	0.470 (0.130–1.696)	0.345
Complete occlusion rate				
6 M	37 (63.8%)	41 (62.1%)	1.384 (0.629–3.042)	0.435
12 M	38 (65.5%)	44 (66.7%)	1.234 (0.549–2.771)	0.684
Recurrence rate				
6 M	3 (5.2%)	2 (3.0%)	1.788 (0.288–11.113)	0.661
12 M	4 (6.9%)	3 (4.5%)	1.595 (0.341–7.457)	0.702
Ischemic stroke				
1 M	1 (1.7%)	3 (4.5%)		0.462
General adverse events				
1 M	9 (15.5%)	11 (16.7%)	0.680 (0.351–2.402)	0.905
6 M	9 (15.5%)	11 (16.7%)	0.680 (0.351–2.402)	0.905
12 M	9 (15.5%)	11 (16.7%)	0.680 (0.351–2.402)	0.905

Data are No. (%). P < 0.05 was considered statistically significant

Statistical analysis showed that complications were comparable between the groups. Overall, general adverse events occurred in 9/58 patients (15.5%) in the Numen group and 11/66 patients (16.7%) in the Axium group, with a common odds ratio of 0.680 (95% CI, 0.351–2.402, P = 0.862). Most of these events were symptoms, such as vomiting, headache, fever, or allergic response. In addition, there were 4 cases of intraoperative ischemic stroke (1 in the Numen group and 3 in the Axium group), which were considered after further examination by MRI and angiography. Among these 4 cases, the case of stroke in the Numen group and one in the Axium group were thought to be caused by parent artery occlusion; the other 2 cases were caused by intrastent thrombosis. All ischemic stroke patients recovered well after medical therapy and rehabilitation.

## Discussion

Although several studies have been conducted to evaluate the safety and efficacy of the endovascular treatment of small intracranial aneurysms, randomized trials are still lacking. We extracted data from the CATCH trial and found that endovascular coiling techniques for treating small intracranial aneurysms are safe and feasible. Our results showed high success rates and complete occlusion immediately after endovascular surgery and during follow-up. According to a meta-analysis by Yamaki et al., technical success was achieved in 92% of patients, and 7% of aneurysms retreated [18]. In our study, the treatment of only one ruptured aneurysm failed. In addition, our results showed lower recurrence rates during long-term follow-up. No patients required retreatment because the mRS score did not increase.

Intraoperative aneurysm rupture is a severe complication in the endovascular treatment of small aneurysms. The small size of the aneurysm sac limits the movement of the microcatheter [20]. Any unexpected move during microcatheter implantation and coil packing can rupture the aneurysm sac. Initial study results show that the intraoperative rupture rate ranged from 9 to 11%. In comparison, recent studies have shown a reduced risk of approximately 3–4% [21, 22]. In our study, there were no intraoperative rupture cases in either group. The difference may be due to newer technological advances and increased experience. The annual number of clinical centers involved in the study is more significant than 300, and surgeons have extensive experience. In addition, technology advances for smaller and softer coils could also have contributed to the results.

This study showed that the Numen coil was non-inferior to the Axium coil in treating small intracranial aneurysms. Unlike Axium coils, there are two types of three-dimensional (3D) coil shapes for Numen coils: 3/4 loops and 1/2 loops. The 3D coil with 3/4 loops is fabricated by a series of Ω-shaped loops, which provides a stable framing in the aneurysm sac. And the 3D coil with 1/2 loops is formed by a series of Ω-shaped and S-shaped loops incorporated for dense filling and safe finishing. The advantage of Numen coils includes: firstly, the anti-unwinding design improves its softness; secondly, the half size standard makes the selection more accurate; thirdly, the shorter caudal segment in length enhances the stability of the microcatheter. The coil packing density was suggested to be reduced, allowing slow thrombus formation in the sac [23, 24]. However, this will increase the risk of postoperative rebleeding.

Although an additional assist device can facilitate coil packing, it may increase the incidence of complications, especially ischemic events [25, 26]. Our results showed a relatively low incidence of intraoperative ischemic stroke, occurring in 4 /124 patients (3.2%). However, none of these four patients received stent- or balloon-assisted coiling treatment. The traditional stents, not flow-diverting stents allow satisfactory angiographic results to be achieved with fewer coils, which may increase the postoperative recurrence rate. The high recurrence rate of stent-assisted coiling may also be related to antiplatelet therapy [27, 28]. Of the seven patients with recurrence, one was treated with the Enterprise stent. Since previous literature has shown that the recurrence rate for aneurysms smaller than 5 mm is low, a significant difference is unlikely to be observed. In this study, there was also no significant difference in the recurrence rate between the groups.

When dealing with small unruptured aneurysms, the natural history of these aneurysms should be considered [29–31]. It has been reported that the annual rupture rates of single and multiple small unruptured aneurysms are only 0.35% and 0.95%, respectively [32]. Before treatment, the risk of yearly rupture should be compared with the risk of surgical complications. Other risk factors should be considered, such as population, hypertension, age, and aneurysm size and shape.

Only small saccular aneurysms were involved in this study, so the results only represent the treatment of one particular type of aneurysm. Well-controlled studies in the future were needed for validation. In addition, although we included aneurysms in the posterior circulation, there were only two cases of such aneurysms. Studies on using these techniques in treating aneurysms in the posterior circulation should be performed.

## Conclusion

Compared with the Aixum coil, the Numen coil is safe and effective in treating small intracranial aneurysms. When treating small intracranial aneurysms, one should consider the natural history of small aneurysms and the risk of treatment before developing an individual surgical plan.

## Data Availability

The datasets used and/or analyzed during the current study are available from the corresponding author on reasonable request.

## Abbreviations

CATCH: Coil Application Trial in China
CEC: Clinical Events Committee
FAS: Full Analysis Set
PPS: Per-Protocol Set
